# More Aβ deposition in left occipital cortex in Cognitive Impairment Patients with Hallucinations

**DOI:** 10.1002/alz70856_100093

**Published:** 2025-12-25

**Authors:** Zhen Qiao, Lin Ai

**Affiliations:** ^1^ Beijing Tiantan Hospital, Beijing, Beijing, China; ^2^ Beijing Tiantan Hospital, Beijing, China

## Abstract

**Background:**

To evaluate the characteristics of Aβ deposition in cognitive impairment patients with hallucinations.

**Method:**

A retrospective analysis was performed on the AV45 PET imaging of 9 cognitive impairment patients with hallucinations (CI‐H) and 9 cognitive impairment patients without hallucinations (CI‐N). The data were analyzed using the cortexID suite. The whole cerebellum was selected as the reference brain region, and the SUVR of each cortical brain region was calculated.

**Result:**

In the CI ‐ H group, there were 5 males and 4 females, aged 59 ‐ 88 years old, with a disease course of 1 ‐ 7 years. In the CI ‐ N group, there were 4 males and 5 females, aged 55 ‐ 82 years old, with a disease course of 1 ‐ 6 years. By visual assessment, 6 cases in the CI ‐ H group had positive AV45 PET imaging and 3 cases were negative. In the CI‐N group, 7 cases were positive and 2 cases were negative. The SUVR of the composite cortex in the CI‐H group was 1.23±0.23, and that in the CI‐N group was 1.23±0.24. In the CI‐H group, 6/9 cases (66.7%) had the highest radiotracer uptake in the occipital cortex (left/right: 5/1), while in the CI‐N group, only 2/9 cases (22.2%) had the highest radiotracer uptake in the occipital cortex. The former was more than the latter, but there was no significant statistical difference. In the CI‐H group, 2 cases had the highest uptake in the left precuneus and posterior cingulate gyrus, and 1 case had the highest uptake in the left lateral temporal cortex. In the CI ‐ N group, 5 cases had the highest uptake in the precuneus and posterior cingulate gyrus (4 cases on left and 1 case on right ), and 2 cases had the highest uptake in the left anterior cingulate gyrus. The SUVR of the occipital lobe in the CI‐H group (L:1.39±0.24 and R:1.33±0.22 ) was higher than that in the CI ‐ N group (L:1.34±0.24 and R:1.29±0.19 ), but there was no significant statistical difference.

**Conclusion:**

Aβ preferentially deposits in the left occipital cortex in cognitive impairment patients with hallucinations.

